# Preoperative Gastric Ultrasonography in Diabetic Versus Non-diabetic Patients Undergoing Elective Surgery: A Prospective Comparative Observational Study of Gastric Volume and Airway Management Implications

**DOI:** 10.7759/cureus.110728

**Published:** 2026-06-12

**Authors:** Sheikh S Ahmed, Shikha Sharma, Nargis Rashid

**Affiliations:** 1 Department of Anesthesiology, Acharya Shri Chander College of Medical Sciences and Hospital, Jammu, IND

**Keywords:** diabetes mellitus, elective surgery, gastric ultrasonography, gastric volume, rapid sequence intubation

## Abstract

Background

Pulmonary aspiration remains a serious perioperative complication associated with considerable morbidity and mortality. Although standard fasting guidelines are intended to minimize aspiration risk, patients with diabetes mellitus may experience delayed gastric emptying despite adequate fasting due to autonomic dysfunction and altered gastric motility. Point-of-care gastric ultrasonography has emerged as a practical bedside tool for evaluating residual gastric contents and may assist in perioperative airway planning. This study aimed to compare fasting gastric volume between diabetic and non-diabetic patients undergoing elective surgery using ultrasonography and to evaluate the distribution of airway management strategies according to preoperative ultrasonographic findings.

Methods

This prospective comparative observational study was conducted in the Department of Anesthesiology and Intensive Care at a tertiary care teaching hospital between June 2024 and November 2025. A total of 122 adult patients of either sex aged 30-75 years undergoing elective surgery under general anesthesia were enrolled and equally divided into Group C (non-diabetic, n = 61) and Group D (diabetic, n = 61). All patients underwent preoperative gastric ultrasonography after at least eight hours of fasting. Gastric antral measurements were obtained in both supine and right lateral decubitus (RLD) positions. Gastric cross-sectional area (CSA) and estimated gastric volume were calculated using standard formulas. Qualitative gastric grading was performed, and airway management strategies were selected according to ultrasonographic findings.

Results

Baseline fasting duration was similar between groups (8.6 ± 0.9 h vs 8.8 ± 0.9 h, p = 0.410). In the supine position, diabetic patients demonstrated significantly larger craniocaudal diameter (2.14 ± 0.14 cm vs 1.92 ± 0.18 cm), anteroposterior diameter (1.35 ± 0.08 cm vs 1.01 ± 0.09 cm), and gastric antral CSA (2.27 ± 0.19 cm^2^ vs 1.48 ± 0.20 cm^2^) compared with non-diabetic patients (p < 0.001 for all). Similar findings were observed in the RLD position, where diabetic patients had larger CSA values (3.34 ± 0.29 cm^2^ vs 1.52 ± 0.21 cm^2^) and significantly greater estimated gastric volume (10.3 ± 13.9 mL vs values consistent with negligible or empty gastric contents in non-diabetic patients; p < 0.001). Grade 0 gastric findings were more frequent among non-diabetic patients (67.2% vs 9.8%), whereas Grade 1 findings were predominant in diabetic patients (83.6% vs 31.1%) (p < 0.001). Rapid sequence intubation was performed more frequently in diabetic patients than non-diabetic patients (19.7% vs 3.3%; p = 0.004).

Conclusions

Diabetic patients demonstrated larger gastric antral dimensions and a higher prevalence of residual gastric contents on qualitative ultrasonographic assessment despite similar fasting duration. Preoperative gastric ultrasonography may help identify residual gastric contents and assist in perioperative airway risk assessment.

## Introduction

Pulmonary aspiration of gastric contents during the perioperative period is an uncommon but potentially life-threatening event associated with considerable morbidity and mortality [1]. Aspiration of gastric contents can result in aspiration pneumonitis, which may progress to acute lung injury, respiratory compromise, and prolonged hospital stay. Because airway protective reflexes become impaired during induction and emergence from general anesthesia, preventing aspiration remains a major concern for anesthesiologists [2].

To reduce this risk, preoperative fasting guidelines have been widely implemented in routine clinical practice [3]. The American Society of Anesthesiologists (ASA) recommends a fasting duration of two hours for clear liquids, four hours for breast milk, and six hours for a light meal before anesthesia administration [4]. These recommendations aim to minimize residual gastric contents at the time of induction. However, these guidelines were primarily developed for healthy individuals and may not adequately address patients with conditions that alter gastrointestinal motility [2].

Certain patient populations are more likely to experience delayed gastric emptying despite following standard fasting recommendations [5]. Diabetes mellitus and obesity are among the most common and rapidly increasing health conditions worldwide that can affect gastric motility [6,7]. In individuals with long-standing or poorly controlled diabetes, autonomic neuropathy may lead to diabetic gastroparesis, a disorder characterized by delayed gastric emptying in the absence of mechanical obstruction [8]. Damage to vagal and enteric neural pathways can impair gastric contractions and disrupt normal coordination between the stomach and pylorus [9]. As a result, diabetic patients may retain significant gastric contents even after an adequate fasting period, increasing the risk of regurgitation and pulmonary aspiration [2].

Despite these concerns, there remains uncertainty regarding the ideal fasting recommendations for diabetic patients. The European Society of Anesthesiology (ESA) fasting guidelines published in 2011 suggested that patients with diabetes may follow fasting instructions similar to those of healthy individuals. In contrast, the ASA highlighted in 2017 that coexisting medical conditions can prolong gastric emptying and may require individualized consideration beyond routine fasting recommendations [10].

In recent years, point-of-care (POC) gastric ultrasonography has emerged as a useful bedside tool in anesthetic practice. It is increasingly used to evaluate gastric contents in situations where fasting status is unclear or in emergency settings where surgery cannot be delayed [11]. Gastric ultrasound offers a noninvasive, real-time assessment of residual gastric volume (GV) and can aid perioperative decision-making. During pre-induction evaluation, this technique may help identify patients with unexpectedly high GVs despite appropriate fasting periods, thereby supporting individualized airway management strategies and reducing aspiration risk [10].

The primary objective of this study was to compare fasting GV and gastric antral cross-sectional area (CSA) between diabetic and non-diabetic patients undergoing elective surgery using gastric ultrasonography. The secondary objectives were to compare qualitative gastric grading and assess the frequency of airway management strategies adopted according to preoperative ultrasonographic findings.

## Materials and methods

Study design and setting

This prospective comparative observational study was conducted in the Department of Anesthesiology and Intensive Care at a tertiary care teaching hospital between June 2024 and November 2025 after obtaining approval from the Institutional Ethics Committee (ASCOMS/IEC/2024/Meeting-II/16).

Study population

A total of 122 adult patients of either sex aged 30-75 years with ASA physical status I-II who were scheduled for elective surgery under general anesthesia were enrolled after obtaining written informed consent. Participants were divided into two groups: Group C included non-diabetic patients (n = 61), while Group D comprised patients with known diabetes mellitus (n = 61). Patients with body mass index (BMI) >30 kg/m^2^, pregnancy, hypothyroidism, previous upper abdominal surgery, use of prokinetic medications, smoking history, or the presence of a nasogastric tube were excluded from the study. Patients were evaluated at a single preoperative time point, and no follow-up or outcome-based participant selection was performed.

Sample size calculation

The sample size was determined using data from Garg et al. [12], which reported significant differences in gastric antral CSA between diabetic and non-diabetic patients. Assuming a study power of 90%, alpha error of 0.05, and an equal allocation ratio, the minimum required sample size was calculated to be 53 patients per group. To improve statistical strength and compensate for potential dropouts, 61 participants were recruited in each group, resulting in a total sample size of 122 patients.

Preoperative assessment

All patients underwent a routine pre-anesthetic evaluation before surgery. All participants followed institutional fasting guidelines, including a minimum fasting period of eight hours for solids and at least two hours for clear liquids before surgery. Clinical details, including demographic data, were recorded. In diabetic patients, duration of diabetes and glycated hemoglobin (HbA1c) levels were recorded as indicators of disease duration and glycemic control.

Gastric ultrasound assessment

Preoperative gastric ultrasonography was performed by a blinded operator using a 2-5 MHz curvilinear probe (FUJIFILM SonoSite Inc., Bothell, WA, USA). The gastric antrum was examined in both the supine and right lateral decubitus (RLD) positions. The gastric antral CSA was calculated using the formula: $CSA = \frac{AP \times CC \times \pi}{4}$, where AP represents the anteroposterior diameter and CC represents the craniocaudal diameter. GV was estimated using the Perlas equation [13]: $GV\ (\mathrm{mL}) = 27.0 + (14.6 \times CSA) - (1.28 \times \mathrm{age})$. For each patient, three consecutive measurements of the antral diameters were obtained in each position, and the average values were used for calculation of CSA and estimated GV.

In addition to quantitative measurements, gastric contents were assessed qualitatively and categorized into four grades. Grade 0 represented an empty stomach, Grade 1 indicated GV less than 1.5 mL/kg, Grade 2 represented GV between 1.5 and 2 mL/kg, and Grade 3 included GV greater than 2 mL/kg or the presence of solid gastric contents. All ultrasonographic measurements were obtained after identification of the gastric antrum using standard anatomical landmarks, including the left lobe of the liver, pancreas, and abdominal aorta/inferior vena cava. All examinations were performed by an anesthesiologist with more than three years of experience in gastric ultrasonography who was blinded to the study objectives during image acquisition and measurements.

Estimated GV per kilogram body weight was calculated by dividing the total estimated GV by body weight (mL/kg). Qualitative grading was subsequently assigned according to the predefined volume thresholds.

Airway management and rapid sequence intubation (RSI)

The airway management strategy was determined according to preoperative gastric ultrasound findings, including estimated GV and qualitative assessment of gastric contents. Patients considered at low risk for aspiration, defined as those with an empty stomach (Grade 0) or GV <1.5 mL/kg, underwent routine intravenous induction followed by standard endotracheal intubation. Patients identified as high risk for aspiration, characterized by GV ≥1.5 mL/kg, qualitative Grade 2 or higher findings, or other features suggestive of significant residual gastric contents, were managed with RSI. In patients with GV >2 mL/kg or suspected solid gastric contents, additional interventions such as aspiration prophylaxis or consideration of postponement of surgery were undertaken at the discretion of the attending anesthesiologist.

A standardized RSI protocol was followed in all eligible patients [14]. Preoxygenation with 100% oxygen was administered for three to five minutes before induction. Intravenous induction was performed using propofol followed by a rapid-acting neuromuscular blocking agent (succinylcholine or rocuronium). Cricoid pressure (Sellick’s maneuver) was applied during induction to reduce the risk of regurgitation. Positive-pressure ventilation before securing the airway was avoided unless clinically necessary, and endotracheal intubation was performed promptly after adequate neuromuscular blockade was achieved.

Statistical analysis

Statistical analysis was performed using IBM SPSS Statistics for Windows, Version 27 (Released 2019; IBM Corp., Armonk, New York, United States). Continuous variables were tested for normality using the Shapiro-Wilk test and were expressed as mean ± standard deviation (SD). Intergroup comparisons for normally distributed continuous variables were performed using the independent samples t-test. Categorical variables were presented as frequencies and percentages and analyzed using the chi-square test or Fisher’s exact test, as appropriate. A p-value of less than 0.05 was considered statistically significant.

## Results

A total of 122 patients were included in the study, with 61 patients each in Group C (non-diabetic) and Group D (diabetic). The mean age was significantly higher among diabetic patients compared to non-diabetic patients (52.0 ± 10.5 years vs. 45.8 ± 13.4 years; p = 0.012). Gender distribution was comparable between the two groups, with males comprising 31 (50.8%) and 29 (47.5%) patients and females comprising 30 (49.2%) and 32 (52.5%) patients in Group C and Group D, respectively (p = 0.717) (Table 1).

**Table 1:** Baseline demographic and clinical characteristics of non-diabetic and diabetic patients undergoing elective surgery. Continuous variables were analyzed using the independent samples t-test, while categorical variables were analyzed using the Chi-square test. *p < 0.05 statistically significant. ASA: American Society of Anesthesiologists

Variable	Group C (non-diabetic) (n = 61)	Group D (diabetic) (n = 61)	Statistics	95% confidence intervals (CI)	p-value
Age (years)	45.8 ± 13.4	52.0 ± 10.5	t = -2.84	-10.52 to -1.88	0.012*
Male	31 (50.8%)	29 (47.5%)	χ^2^ = 0.131	-	0.717
Female	30 (49.2%)	32 (52.5%)			
Height (cm)	163.7 ± 7.8	162.9 ± 8.1	t = 0.56	-2.05 to 3.65	0.590
Weight (kg)	64.3 ± 6.8	67.2 ± 7.5	t = -2.24	-5.47 to -0.33	0.046*
BMI (kg/m^2^)	23.9 ± 2.0	25.3 ± 2.1	t = -3.77	-2.14 to -0.66	0.004*
ASA I	35 (57.4%)	0	χ^2^ = 49.08	-	<0.001*
ASA II	26 (42.6%)	61 (100%)			
Fasting duration (h)	8.6 ± 0.9	8.8 ± 0.9	t = -1.23	-0.52 to 0.12	0.410
Duration of diabetes (years)	-	9.2 ± 4.1	-	-	-
HbA1c (%)	5.2 ± 0.4	7.4 ± 1.1	t= -14.68	-2.50 to -1.90	<0.001*

The mean height was similar between both groups, with values of 163.7 ± 7.8 cm in Group C and 162.9 ± 8.1 cm in Group D (p = 0.590). However, diabetic patients had significantly greater body weight (67.2 ± 7.5 kg vs. 64.3 ± 6.8 kg; p = 0.046) and higher BMI values (25.3 ± 2.1 kg/m^2^ vs. 23.9 ± 2.0 kg/m^2^; p = 0.004) compared with non-diabetic patients (Table 1).

Regarding ASA physical status, 35 (57.4%) patients in Group C belonged to ASA I and 26 (42.6%) to ASA II. In contrast, all patients in Group D were classified as ASA II (61 (100%)), resulting in a statistically significant difference between groups (p < 0.001). Mean fasting duration was comparable between the groups, measuring 8.6 ± 0.9 hours in Group C and 8.8 ± 0.9 hours in Group D (p = 0.410) (Table 1).

Among diabetic patients, the mean duration of diabetes was 9.2 ± 4.1 years. HbA1c levels were significantly higher in Group D compared with Group C (7.4 ± 1.1% vs. 5.2 ± 0.4%; p < 0.001), reflecting poorer glycemic control among diabetic individuals (Table 1).

In the supine position, diabetic patients demonstrated significantly larger gastric antral dimensions compared with non-diabetic patients. The mean CC diameter was significantly higher in Group D than Group C (2.14 ± 0.14 cm vs. 1.92 ± 0.18 cm; p < 0.001). Similarly, the mean AP diameter was greater among diabetic patients (1.35 ± 0.08 cm vs. 1.01 ± 0.09 cm; p < 0.001) (Table 2).

**Table 2:** Gastric antrum dimensions in the supine position among non-diabetic and diabetic patients. P-values were calculated using the independent samples t-test. *p < 0.05 statistically significant. CC: craniocaudal diameter; AP: anteroposterior; CSA: cross-sectional area

Parameter	Group C (mean ± SD)	Group D (mean ± SD)	t-value	95% CI of difference	p-value
CC diameter (cm)	1.92 ± 0.18	2.14 ± 0.14	-7.50	-0.28 to -0.16	<0.001*
AP diameter (cm)	1.01 ± 0.09	1.35 ± 0.08	-21.94	-0.37 to -0.31	<0.001*
CSA (cm^2^)	1.48 ± 0.20	2.27 ± 0.19	-22.39	-0.86 to -0.72	<0.001*

The mean gastric antral CSA was also significantly larger in diabetic patients compared with non-diabetic patients (2.27 ± 0.19 cm^2^ vs. 1.48 ± 0.20 cm^2^; p < 0.001). These findings suggest increased gastric antral size among diabetic individuals despite preoperative fasting, indicating a tendency toward greater residual gastric contents in this population (Table 2).

In the RLD position, diabetic patients had significantly larger gastric antral measurements compared with non-diabetic patients. The mean CC diameter was significantly higher in Group D than Group C (2.41 ± 0.12 cm vs. 1.88 ± 0.07 cm; p < 0.001). Likewise, the mean AP diameter was greater in diabetic patients (1.75 ± 0.10 cm vs. 1.03 ± 0.13 cm; p < 0.001) (Table 3).

**Table 3:** Gastric antrum dimensions and estimated gastric volume in the right lateral decubitus (RLD) position among non-diabetic and diabetic patients. P-values were calculated using the independent samples t-test. *p < 0.05 statistically significant. CC: craniocaudal diameter; AP: anteroposterior; CSA: cross-sectional area

Parameter	Group C (mean ± SD)	t-value	95% CI of difference	Group D (mean ± SD)	p-value
CC diameter (cm)	1.88 ± 0.07	-29.65	-0.57 to -0.49	2.41 ± 0.12	<0.001*
AP diameter (cm)	1.03 ± 0.13	-34.34	-0.76 to -0.68	1.75 ± 0.10	<0.001*
CSA (cm^2^)	1.52 ± 0.21	-39.60	-1.91 to -1.73	3.34 ± 0.29	<0.001*
Gastric volume (mL)	-8.7 ± 19.8	-6.18	-25.09 to -12.91	10.3 ± 13.9	<0.001*

The mean CSA was also markedly higher in Group D compared with Group C (3.34 ± 0.29 cm^2^ vs. 1.52 ± 0.21 cm^2^; p < 0.001). Similarly, estimated GV was significantly greater in diabetic patients (10.3 ± 13.9 mL vs. -8.7 ± 19.8 mL; p < 0.001). These findings indicate that diabetic patients had greater residual gastric contents despite fasting, which may reflect delayed gastric emptying and a potentially increased risk of aspiration (Table 3).

These values result from application of the prediction model in patients with very small gastric antral CSAs and should be interpreted as indicating negligible gastric contents rather than true negative GVs.

Qualitative ultrasonographic assessment showed significant differences in gastric content grading between the two groups (χ^2^ = 42.49, p < 0.001). Grade 0 findings, representing an empty stomach, were observed more frequently in non-diabetic patients, with 41 (67.2%) patients in Group C compared with only six (9.8%) patients in Group D (Table 4).

**Table 4:** Qualitative ultrasonographic grading of gastric contents among non-diabetic and diabetic patients. P-values were calculated using the chi-square test. *p < 0.05 statistically significant.

Grade	Group C (n = 61)	Group D (n = 61)	Statistics
Grade 0	41 (67.2%)	6 (9.8%)	χ^2^ = 42.49; p < 0.001*
Grade 1	19 (31.1%)	51 (83.6%)	
Grade 2	1 (1.6%)	4 (6.6%)	
Grade 3	0	0	

Grade 1 findings were more common among diabetic patients and were observed in 51 (83.6%) patients in Group D compared with 19 (31.1%) patients in Group C. Grade 2 findings were relatively uncommon and were seen in only one (1.6%) patient in Group C and four (6.6%) patients in Group D. No patient in either group demonstrated Grade 3 findings. These observations suggest a higher prevalence of residual gastric contents among diabetic patients despite adherence to fasting recommendations (Table 4).

A significant difference was observed in the type of intubation technique used between the two groups (χ^2^ = 8.069, p = 0.004). Routine endotracheal intubation was performed in the majority of patients in both groups; however, it was more frequently used in non-diabetic patients, accounting for 59 (96.7%) patients in Group C compared with 49 (80.3%) patients in Group D (Table 5).

**Table 5:** Comparison of intubation techniques between non-diabetic and diabetic patients. P-values were calculated using the chi-square test. *p < 0.05 statistically significant. RSI: rapid sequence intubation

Intubation type	Group C (n = 61)	Group D (n = 61)	Statistics
Routine	59 (96.7%)	49 (80.3%)	χ^2^ = 8.069; p = 0.004*
RSI	2 (3.3%)	12 (19.7%)	

RSI was required more often among diabetic patients, being performed in 12 (19.7%) patients in Group D compared with only two (3.3%) patients in Group C. The higher frequency of RSI among diabetic individuals reflected the predefined airway management protocol based on ultrasonographic findings (Table 5).

No episodes of pulmonary aspiration, regurgitation, oxygen desaturation related to aspiration, or other major airway-related complications were observed in either group. Furthermore, no surgeries were postponed, cancelled, or delayed based on gastric ultrasonography findings.

## Discussion

The present study found that diabetic patients had significantly larger fasting gastric antral dimensions and a higher prevalence of residual gastric contents on qualitative ultrasonographic assessment compared with non-diabetic patients despite similar fasting durations. These findings suggest that diabetic patients may demonstrate a higher prevalence of residual gastric contents despite adherence to standard preoperative fasting guidelines. The higher residual GVs observed among diabetic patients may be associated with altered gastric motility and diabetic autonomic dysfunction reported in previous studies [15,16]. Long-standing hyperglycemia may affect vagal nerve function and disrupt gastric contractions, resulting in slower gastric emptying and increased residual gastric contents [8].

The findings of the present study are in agreement with previous studies. Garg et al. [12] reported significantly greater gastric antral CSA and GV in diabetic patients compared with non-diabetic controls. Similar observations were reported by Haramgatti et al. [17] and Paidimuddala et al. [10], who also found higher residual gastric contents among diabetic individuals despite adherence to fasting guidelines. In the present study, the mean CSA in the RLD position was 3.34 cm^2^ in diabetic patients compared with 1.52 cm^2^ in non-diabetic patients, which is comparable to findings reported in earlier studies [12,17]. The consistency of these findings across different studies supports the reliability and reproducibility of the observed association between diabetes and delayed gastric emptying.

However, findings from previous studies have not been entirely uniform. Perlas et al. [13] conducted a non-inferiority study and reported no significant difference in fasting GVs between diabetic and non-diabetic patients. Variations in study design and patient characteristics may explain these differing results. In the present study, diabetic patients had a mean disease duration of 9.2 ± 4.1 years and elevated HbA1c values, suggesting relatively prolonged disease duration and suboptimal glycemic control. Previous studies have shown that longer disease duration and poor glycemic control are associated with greater autonomic dysfunction and a higher risk of diabetic gastroparesis [15,16]. These factors may be associated with the larger residual GVs observed in our study population.

An important aspect of the present study was the use of gastric ultrasound findings to guide airway management decisions. Earlier studies mainly focused on validating gastric ultrasonography as a tool for estimating GV [13,18]. In contrast, the present study evaluated the distribution of airway management strategies according to predefined ultrasonographic findings. Based on ultrasound findings, RSI was required in 19.7% of diabetic patients compared with only 3.3% of non-diabetic patients. These findings demonstrate differences in airway management strategies applied according to predefined ultrasonographic criteria. However, because airway management decisions were protocol-driven, the observed difference in RSI rates should not be interpreted as an independent clinical outcome.

The findings also have important clinical implications. Standard fasting duration alone may not accurately reflect gastric emptying status in diabetic patients. Although most participants remained within acceptable GV limits, a proportion of diabetic patients had increased GVs despite adequate fasting. Identifying such patients before induction of anesthesia may allow anesthesiologists to adopt preventive measures such as RSI, application of cricoid pressure, or postponement of surgery when clinically appropriate. This supports a more individualized approach to perioperative care.

Interestingly, no patient in either group demonstrated Grade 3 gastric contents, indicating that standard fasting protocols remain effective for most patients. Nevertheless, diabetic patients showed a substantially higher frequency of residual fluid-containing stomachs compared with non-diabetic individuals. Therefore, gastric ultrasonography should be considered a useful adjunct to fasting guidelines rather than a substitute for them.

Major strengths of the present study include its prospective design, equal group allocation, standardized ultrasonographic assessment, and evaluation of airway management strategies applied according to preoperative ultrasonographic findings.

The present study has certain limitations. First, although the calculated sample size requirement was achieved, the study was conducted at a single tertiary care center with a moderate sample size, which may limit the generalizability of the findings. Second, gastric ultrasonography is an operator-dependent technique; all measurements were obtained by a single operator, and interobserver variability was not assessed. Averaging multiple independent measurements may have improved measurement precision. Third, actual perioperative aspiration events were not evaluated, and airway management decisions were based primarily on ultrasonographic findings rather than clinical outcomes. Additionally, diabetic autonomic neuropathy and the severity of gastroparesis were not objectively assessed, which may have influenced gastric emptying patterns among diabetic patients. Potential confounding factors such as BMI, ASA physical status, duration of diabetes, and glycemic control were not adjusted for in multivariable analysis and may have influenced the observed findings. Diabetic patients were significantly older and had greater body weight and BMI than non-diabetic patients. As age is incorporated into the GV prediction model, these baseline differences may have contributed to the observed between-group differences. The absence of multivariable adjustment limits the ability to determine whether the observed findings were independently associated with diabetes. Furthermore, the higher frequency of RSI observed among diabetic patients should be interpreted cautiously because airway management strategies were predetermined according to ultrasonographic findings and were not independent clinical outcomes. Although negative GV estimates were generated in some patients through application of the prediction model, such values are not physiologically meaningful and should be interpreted as representing negligible or empty gastric contents. Finally, the use of medications known to influence gastric emptying, such as glucagon-like peptide-1 (GLP-1) receptor agonists, was not specifically recorded and may have affected residual GV measurements.

Future studies should include larger and more diverse patient populations and evaluate additional factors such as age, BMI, duration of diabetes, HbA1c levels, and autonomic dysfunction through multivariable analysis to identify independent predictors of delayed gastric emptying. Long-term studies examining the relationship between ultrasonographic findings and actual perioperative aspiration events may provide further evidence regarding the clinical significance of these observations.

## Conclusions

Diabetic patients undergoing elective surgery demonstrated larger gastric antral dimensions and a higher prevalence of residual gastric contents on qualitative ultrasonographic assessment compared with non-diabetic patients despite similar fasting durations. These findings indicate an association between diabetes and residual gastric contents; however, the observational design and baseline differences between groups preclude causal inference. A greater proportion of diabetic patients underwent RSI according to predefined ultrasonographic criteria. Further studies incorporating multivariable adjustment and clinical outcome measures are needed to better define the relationship between diabetes, gastric emptying, and perioperative aspiration risk.
